# Rapamycin Dampens Inflammatory Properties of Bone Marrow ILC2s in IL-33-Induced Eosinophilic Airway Inflammation

**DOI:** 10.3389/fimmu.2022.915906

**Published:** 2022-06-03

**Authors:** Emma Boberg, Julie Weidner, Carina Malmhäll, Jenny Calvén, Carmen Corciulo, Madeleine Rådinger

**Affiliations:** ^1^ Krefting Research Centre, Department of Internal Medicine and Clinical Nutrition, Institute of Medicine, Sahlgrenska Academy, University of Gothenburg, Gothenburg, Sweden; ^2^ Centre for Bone and Arthritis Research, Department of Rheumatology and Inflammation Research, Institute of Medicine, Sahlgrenska Academy, University of Gothenburg, Gothenburg, Sweden

**Keywords:** innate lymphoid cells, interleukin-33, rapamycin, interleukin-5, eosinophils, bone marrow, mTOR, asthma

## Abstract

The alarmin cytokine interleukin (IL)-33 plays an important proinflammatory role in type 2 immunity and can act on type 2 innate lymphoid cells (ILC2s) and type 2 T helper (T_H_2) cells in eosinophilic inflammation and asthma. The mechanistic target of rapamycin (mTOR) signaling pathway drives immune responses in several inflammatory diseases, but its role in regulating bone marrow responses to IL-33 is unclear. The aim of this study was to determine the role of the mTORC1 signaling pathway in IL-33-induced bone marrow ILC2 responses and its impact on IL-33-induced eosinophilia. Wild-type mice were intranasally exposed to IL-33 only or in combination with the mTORC1 inhibitor, rapamycin, intraperitoneally. Four groups were included in the study: saline-treated (PBS)+PBS, rapamycin+PBS, PBS+IL-33 and rapamycin+IL-33. Bronchoalveolar lavage fluid (BALF), serum and bone marrow cells were collected and analyzed by differential cell count, enzyme-linked immunosorbent assay and flow cytometry. IL-33 induced phosphorylation of the mTORC1 protein rpS6 in bone marrow ILC2s both *ex vivo* and *in vivo*. The observed mTOR signal was reduced by rapamycin treatment, indicating the sensitivity of bone marrow ILC2s to mTORC1 inhibition. IL-5 production by ILC2s was reduced in cultures treated with rapamycin before stimulation with IL-33 compared to IL-33 only. Bone marrow and airway eosinophils were reduced in mice given rapamycin before IL-33-exposure compared to mice given IL-33 only. Bone marrow ILC2s responded to IL-33 *in vivo* with increased mTORC1 activity and rapamycin treatment successfully decreased IL-33-induced eosinophilic inflammation, possibly by inhibition of IL-5-producing bone marrow ILC2s. These findings highlight the importance of investigating specific cells and proinflammatory pathways as potential drivers of inflammatory diseases, including asthma.

## 1 Introduction

While type 2 inflammation and eosinophilia are typically associated with allergic asthma, they can also be present during non-allergic asthma. The mechanisms behind non-allergic asthma are not fully understood, though it is believed to be caused by an altered airway epithelium response to environmental factors. Interleukin (IL)-33 is released from the airway epithelium when triggered by environmental factors, including allergens (1). Thus, IL-33, a member of the IL-1 family, is an important inflammatory driver in eosinophilic inflammation and asthma (2–5). IL-33 signaling is involved in both allergic and non-allergic eosinophilic inflammation. This includes type 2 cytokine production by type 2 innate lymphoid cells (ILC2s) and T helper 2 (T_H_2) cells, both of which express the IL-33 receptor ST2 (6–11). Mouse lung ILC2s play a central role in worsening antigen-induced inflammatory responses mediated by IL-33 (10). Higher expression of IL-33 is associated with asthma severity, and IL-33 is considered a promising therapeutic target for eosinophilic asthma (2, 12, 13). Indeed, patients with eosinophilic asthma display elevated serum levels of IL-33 compared to non-eosinophilic phenotypes (14).

Eosinophils develop from progenitor cells in the bone marrow, and IL-5 is crucial for cell migration, terminal differentiation, and proliferation (7, 15, 16). ILC2s are potent producers of type 2 cytokines, including IL-5, and play important roles in several diseases, including asthma (2, 17–19). We have previously identified bone marrow ILC2s as an early source of IL-5 after IL-33 challenge, demonstrating that bone marrow ILC2s are important mediators central to eosinophil development (20). In addition, we have shown in two different IL-33-dependent murine models of allergic airway inflammation, i.e. papain and house dust mite (HDM), that bone marrow ILC2s are IL-33-responsive cells (9, 21). Furthermore, we demonstrated that the adaptive immune system was dispensable during both papain challenge and IL-33-induced eosinophilic airway inflammation, exhibiting ILC2s as potent sources of type 2 cytokines driving the eosinophilic inflammation. However, the mechanisms regulating the inflammatory properties of bone marrow ILC2s in IL-33-induced inflammation are still poorly understood.

The mechanistic target of rapamycin (mTOR) signaling pathway regulates many cellular processes including cell metabolism, migration, differentiation and cytokine responses (22, 23). The mTOR signaling pathway is implicated in several inflammatory diseases (23–27) and rapamycin, an inhibitor of mTOR Complex 1 (mTORC1), can suppress eosinophil differentiation in both allergic and non-allergic airway inflammation (28–30). Zhang et al. reported that eosinophil infiltration in mouse lung was impaired after treatment with rapamycin and other mTOR inhibitors in an ovalbumin-induced asthma model (31). Furthermore, the same group showed increased mTOR levels in serum from children with asthma exacerbations compared to patients in asthma remission (31). Another murine study reported decreased eosinophilia when rapamycin was administered together with HDM, but worsened inflammation when rapamycin was administered during ongoing eosinophilic inflammation (28). Thus, the effects of mTORC1 inhibition on eosinophilic inflammation remain unclear.

Accumulating evidence indicates that ILCs have tissue-specific functions (32). ILC2s exhibit different phenotypes and functions at different localizations, and single-cell profiling identified ILC2 subsets that expressed distinct activating receptors (33). Salmond et al. reported an important role for the mTOR signaling pathway in the inflammatory functions of lung ILC2s and CD4^+^ T_H_2 cells during IL-33-induced eosinophilic inflammation (30). However, the role of mTOR in the regulation and function of bone marrow ILC2s remains elusive. Thus, the overall aim of this study was to determine the role of the mTORC1 signaling pathway *in vivo* in response to IL-33 in bone marrow ILC2s. We further investigated whether mTORC1 inhibition, by rapamycin treatment, modifies inflammatory properties of bone marrow ILC2s in IL-33-induced eosinophilic inflammation.

## 2 Materials and Methods

### 2.1 Mice

Wild-type (WT) mice, C57BL/6J were purchased from Charles River (Sulzfeld, Germany) or obtained *via* in-house breeding (University of Gothenburg, Sweden). Male mice used in all experiments were 10 – 12 weeks old, housed in pathogen-free conditions and given food and water *ad libitum*. All animal experiments were approved by the Gothenburg County Regional Ethical Committee (permit numbers 126/14 and 2459/19).

### 2.2 *In Vivo* Model

WT mice were given 1 µg recombinant murine (rm) IL-33 (PeproTech, Rocky Hill, NJ, USA) intranasally (i.n.) every other day for a total of 5 days. Rapamycin doses (2 mg/kg, R0395, Sigma-Aldrich, St. Louis, MO, USA, dissolved in dimethyl sulfoxide) were administered intraperitoneally (i.p.) one hour before each i.n. IL-33 administration. Control mice received phosphate buffered saline (PBS) vehicle i.n. and i.p., where the i.p. injection contained the same concentration of dimethyl sulfoxide for mice receiving rapamycin. Four groups were included in the study: PBS+PBS, rapamycin+PBS, PBS+IL-33, and rapamycin+IL-33, where the former in each group describes the i.p. dosing and the latter describes the i.n. dosing.

### 2.3 Sample Procedures

Serum, bronchoalveolar lavage fluid (BALF) and bone marrow cells were collected 24 h after the final exposure. Blood was obtained by puncturing the heart. BALF was collected by instilling 0.25 mL of PBS through the tracheal cannula, followed by gentle aspiration and a second lavage of 0.25 mL. BALF cells were processed for differential cell count analysis and cell-free BALF and serum were processed for mediator analysis by enzyme-linked immunosorbent assay (ELISA). Bone marrow cells were obtained from left and right femurs flushed with 5 mL wash buffer each (2% fetal bovine serum in 1xPBS) and filtered through a 100 µm cell strainer (CellTrics^®^, Sysmex, Goerlitz, Germany). Bone marrow cells were then processed for differential cell count, *ex vivo* stimulations and flow cytometry. The red blood cells were lysed using red blood cell lysis buffer (0.1 mM EDTA in distilled water/0.8% NH_4_Cl, Sigma-Aldrich/Merck Chemicals) and incubated for 10 min on ice.

### 2.4 Mediator Measurements

Concentrations of serum IL-5 and BALF CCL24/eotaxin-2 were measured using mouse ELISA DuoSets (R&D Systems, Minneapolis, MN) according to the manufacturer’s instructions. The BM Chemiluminescence ELISA (POD) substrate kit (Roche Diagnostics GmbH, Roche Applied Science, Mannheim, Germany) was used for detection of signal and luminescence was measured on a Varioskan™ LUX multimode microplate reader (ThermoFisher Scientific, Vantaa, Finland). Samples below the detection limit were set to zero.

### 2.5 Differential Cell Count

Approximately 10,000-50,000 cells were collected via cytospin (425 x g, 6 min, Shandon Cytospin 3 centrifuge) and stained with Hemacolor^®^ Rapid stain (Merck, Darmstadt, Germany) according to the manufacturer’s protocol. Eosinophils were assessed under an Axioplan 2 microscope (Carl Zeiss Jena GmbH).

### 2.6 *Ex Vivo* Stimulation of Bone Marrow Cells With Rapamycin and IL-33 for Intracellular Analysis

#### 2.6.1 mTORC1 Activity in ILC2s and T_H_ Cells

To determine if the mTORC1 signaling pathway was activated in ILC2s and T_H_ cells, bone marrow cells from naïve WT mice were incubated for one hour with rapamycin (50 ng/mL). Cells were stimulated with rmIL-33 (100 ng/mL) for 15 minutes. Complete cell medium was used as a control, containing RPMI-1640 (HyClone™; GE Healthcare Life Sciences, South Logan, UT, USA), 10% fetal bovine serum (Sigma-Aldrich), 2 mM L-glutamine (HyClone), 100 U/mL penicillin, 100 μg/mL streptomycin (HyClone), 1 mM sodium pyruvate (Sigma-Aldrich). Phosphorylated ribosomal protein rpS6 (pRPS6), an mTORC1 target, was measured by intracellular flow cytometry.

#### 2.6.2 Intracellular IL-5 Measurements in ILC2s

A total of 4 x 10^6^ bone marrow cells from naïve WT mice were incubated for one hour with rapamycin (50 ng/mL), followed by 3 h stimulation with rmIL-33 (100 ng/mL). Monensin (BD GolgiStop™, BD Biosciences, Erembodegem, Belgium) was added to all samples (4 μL GolgiStop™/6 mL media) before IL-33 stimulation. The frequency of IL-5^+^ ILC2s was measured by intracellular flow cytometry.

### 2.7 Flow Cytometry

#### 2.7.1 Surface Staining

Bone marrow cells were resuspended in 2% mouse serum (Dako, Glostrup, Denmark) and surface antibodies were added (30 min, 4°C, in the dark). For *ex vivo* experiments, cells were also stained with viability dye (Live/Dead™Fixable Aqua stain, Invitrogen, Life Technologies Corp, Eugene, Oregon, USA). Cells were washed, then fixed (BD CellFix^™^, BD Biosciences) for 15 min in the dark at room temperature and washed before intracellular staining or direct analysis. For intracellular staining, monensin (4 µL GolgiStop™/6 mL buffer) was added to all solutions prior to fixation.

#### 2.7.2 Intracellular Staining of IL-5

After fixation, cells were washed and permeabilized using 0.1% saponin (Sigma-Aldrich) in Hank’s balanced salt solution. Anti-IL-5 antibodies or isotype control antibodies (**Table S1**) were added and incubated 40 min (in the dark, at room temperature) followed by washing and flow cytometric analysis.

#### 2.7.3 Phospho-Protein Analysis of ILC2s and T_H_ Cells

Bone marrow cells were resuspended in 2% mouse serum (Dako) and surface antibodies were added (30 min, 4°C). Cells were washed and fixed (BD Cytofix™, BD Biosciences) for 10 min in the dark at 37°C. Cells were washed and permeabilized using ice-cold Phosflow™ Perm Buffer II (BD Biosciences) followed by 30 min incubation on ice. Cells were washed, stained for intracellular anti-S6 (60 min in the dark, at room temperature, **Table S1**), and washed again before analysis.

#### 2.7.4 Analysis

Flow cytometric analysis was performed using a BD FACSVerse™ flow cytometer running BD FACSuite™ version 1.0.6. Collected data were analyzed by FlowJo™ software (BD Biosciences). Eosinophil progenitors and mature eosinophils were defined as SSC^lo^CD45^+^CD34^+^CD125^+^ and SSC^hi^CD45^+^CD34^-^CD125^lo^CCR3^+^Siglec-F^+^ respectively. T_H_ cells were defined as SSC^lo^CD45^+^CD3^+^CD4^+^CD8^-^B220^-^. Lineage-negative cells were defined as CD3^-^CD45R/B220^-^CD11b^-^TER-119^-^Ly-G6/Gr1^-^CD11c^-^CD19^-^NK-1.1^-^FceR1^-^. ILC2s were defined as SSC^lo^Lin^-^CD45^+^CD127^+^CD25^+^ST2^+^. Antibodies used are listed in **Table S1**. The ST2 expression on ILC2s, chemokine receptor 3 (CCR3) expression on SSC^hi^CD45^+^CD34^-^CD125^lo^, and the intensity of IL-5 and pRPS6 in ILC2s were estimated by mean fluorescence intensity (MFI) values. Relative MFI (rMFI) equals MFI of monoclonal antibody divided by MFI of corresponding fluorescence minus one value.

### 2.8 Statistical Analysis

Data are expressed as mean ± SEM and Graphpad Prism 8 Software (Graphpad Software Inc., La Jolla, CA, USA) was used for statistical analysis. Paired Student’s t-test was used for the *ex vivo* experiments, and Mann-Whitney U test was used for *in vivo* comparisons. Statistical significance was defined as *p<0.05, **p<0.01, ***p<0.001 and ****p<0.0001.

## 3 Results

### 3.1 Rapamycin Treatment Reduced IL-33-Induced Bone Marrow and Airway Eosinophilia

**Figure 1:**
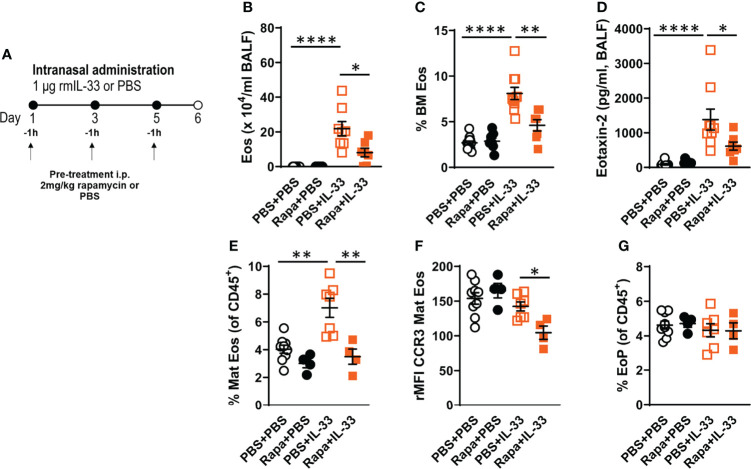
Rapamycin reduced IL-33-induced eosinophilia in BALF and bone marrow. **(A)** C57BL/6 male mice received rapamycin or PBS i.p. one hour before intranasal challenges of rmIL-33 or PBS. Analysis was performed 24 h after the final challenge. **(B)** Number of eosinophils in BALF and **(C)** bone marrow assessed by differential cell count. **(D)** CCL24/Eotaxin-2 concentration in BALF quantified by ELISA. **(E)** Number of mature eosinophils among all CD45^+^ leukocytes in the bone marrow. **(F)** CCR3 expression on mature eosinophils measured by relative mean fluorescence intensity (rMFI). **(G)** Number of eosinophil progenitors among all CD45^+^ leukocytes in the bone marrow. Data are representative of 1 – 3 independent experiments (4 – 12/group) and displayed as mean ± SEM. Mann-Whitney U test. *P < 0.05, **P < 0 .01 and ****P < 0.0001. Eos, Eosinophil; BM, Bone marrow; Mat Eos, Mature eosinophil; EoP, Eosinophil progenitor; Rapa, Rapamycin; i.p, intraperitoneal; i.n., intranasal; PBS+PBS, PBS i.p. and PBS i.n; Rapa+PBS, Rapa i.p. and PBS i.n.; PBS+IL-33, PBS i.p. and IL-33 i.n.; Rapa+IL-33, Rapa i.p. and IL-33 i.n.

To determine the role of rapamycin in eosinophilia, we administered rapamycin to C57BL/6 male mice before inducing eosinophilic inflammation by IL-33 (**Figure 1A**). As expected, IL-33 induced airway and bone marrow eosinophilia (**Figures 1B, C**) and elevated CCL24/eotaxin-2 levels (**Figure 1D**) in BALF compared to control groups. Reduced levels of airway and bone marrow eosinophils and lower concentrations of CCL24/eotaxin-2 were observed in IL-33-exposed mice given rapamycin versus IL-33-exposure alone. Analysis of bone marrow eosinophils by flow cytometry revealed an increase of mature eosinophils in mice exposed to rmIL-33 compared to control mice given PBS. Mice treated with rapamycin before IL-33-exposure exhibited normal levels of mature eosinophils, i.e., similar numbers as control mice (**Figure 1E**). Furthermore, the expression of the chemokine receptor CCR3, which is highly expressed on eosinophils and a receptor for eotaxins, was analyzed on eosinophils. The CCR3 expression on mature eosinophils was significantly reduced in the rapamycin-treated and IL-33-exposed group, suggesting impaired recruitment of eosinophils to the airways (**Figure 1F**). No difference in eosinophil progenitor numbers or expression of the IL5Rα were seen among the treatment groups (**Figures 1G**, **S1**).

### 3.2 The mTORC1 Signaling Pathway Is Critical for IL-5 Production by Bone Marrow ILC2s

**Figure 2:**
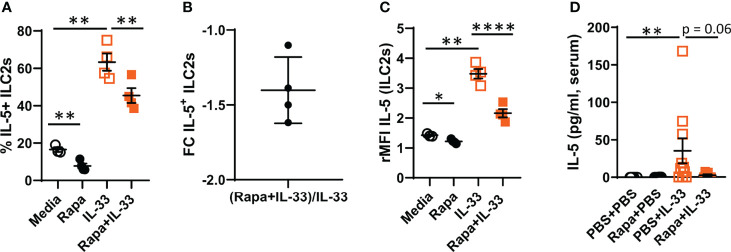
Decreased IL-5 production by bone marrow ILC2s after rapamycin treatment and IL-33 stimulation *ex vivo*. **(A)** Number of IL-5^+^ ILC2 cells. **(B)** Number of IL-5^+^ ILC2s in rapamycin+IL-33 and PBS+IL-33 mice shown as fold change (FC). **(C)** IL-5 intensity in ILC2s measured by relative mean fluorescence intensity (rMFI). **(D)** Concentration of IL-5 in serum. Data are representative of 1 – 3 independent experiments (3 – 10/group). Paired Student’s t-test **(A ,C)**, Mann-Whitney U test **(D)**. *P < 0.05, **P < 0.01 and ****P < 0.0001. Rapa, Rapamycin; i.p, intraperitoneal; i.n., intranasal; PBS+PBS, PBS i.p. and PBS i.n; Rapa+PBS, Rapa i.p. and PBS i.n.; PBS+IL-33, PBS i.p. and IL-33 i.n.; Rapa+IL-33, Rapa i.p. and IL-33 i.n.

We have previously demonstrated that during IL-33-driven inflammation, bone marrow ILC2s produce IL-5, a critical cytokine for eosinophil differentiation (20, 21). In the current study, we found a decrease in IL-5-producing bone marrow ILC2s in cultures treated with rapamycin before rmIL-33 compared to cultures stimulated with IL-33 only (**Figures 2A, B**). Additionally, rMFI revealed decreased overall intensity of IL-5 in ILC2s after pre-treatment with rapamycin (**Figure 2C**). *In vivo*, we found a lower concentration of IL-5 in serum from mice pre-treated with rapamycin before IL-33 exposure compared to mice treated with PBS before IL-33 exposure (**Figure 2D**).

### 3.3 IL-33 Induces mTORC1 Activity in Bone Marrow ILC2s *Ex Vivo*

**Figure 3:**
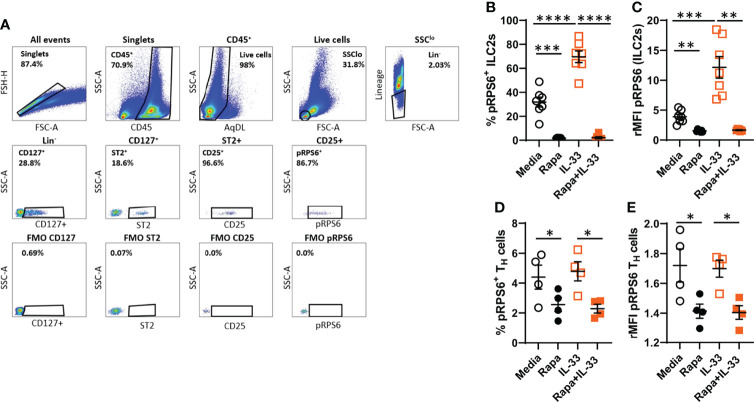
IL-33 induced high mTORC1 activity in bone marrow ILC2s *ex vivo*. **(A)** Gating strategy for quantifying mTOR activity in ILC2s measured by pRPS6 after IL-33 stimulation of cultured bone marrow cultures. **(B)** Number of pRPS6^+^ ILC2s and **(C)** pRPS6 intensity in ILC2s measured by relative mean fluorescence intensity (rMFI). **(D)** Number of pRPS6^+^ T_H_ cells and **(E)** pRPS6 intensity in T_H_ cells measured rMFI. Data are representative of 1 – 2 independent experiments (4 – 7/group). Paired Student’s t-test. *P < 0.05, **P < 0 .01, ***P < 0.001 and ****P < 0.0001. Rapa, Rapamycin.

To determine the mTORC1 activity in bone marrow ILC2s after IL-33 stimulation, we measured phosphorylation of the known mTORC1 target rpS6 by intracellular flow cytometry. The gating strategy for bone marrow pRPS6^+^ILC2s is shown in **Figure 3A**. IL-33-stimulation of bone marrow cultures induced phosphorylation of rpS6^+^ ILC2s compared to controls (**Figure 3B**). Rapamycin inhibited the basal pRPS6^+^ ILC2s signal compared to media only (**Figure 3B**). The overall intensity of pRPS6 in bone marrow ILC2s was increased after IL-33 stimulation compared to controls. This effect was inhibited in cultures treated with rapamycin in addition to IL-33 (**Figure 3C**). Because ILCs share some immune functions with T cells, we also examined mTORC1 activity in bone marrow T_H_ cells compared to ILC2s. IL-33 did not induce rpS6 phosphorylation in bone marrow T_H_ cells compared to controls (**Figure 3D**). Additionally, controls showed that only a small percentage of T_H_ cells were pRPS6^+^ at baseline and that rapamycin further reduced this basal activity (**Figures 3D, E**).

### 3.4 IL-33 Induces mTORC1 Activity in Bone Marrow ILC2s *In Vivo*

**Figure 4:**
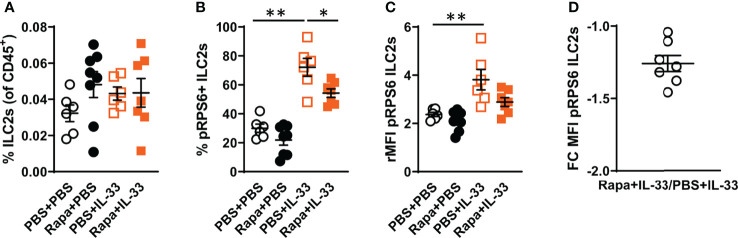
IL-33 induced high mTORC1 activity in bone marrow ILC2s *in vivo*. **(A)** Number of ILC2s among all CD45^+^ leukocytes in the bone marrow. **(B)** Number of pRPS6^+^ ILC2s and **(C)** pRPS6 intensity in ILC2s measured by relative mean fluorescence intensity (rMFI) in mice exposed to IL-33+rapamycin or PBS. **(D)** Fold change **(FC)** rMFI pRPS6 in mice exposed to IL-33+rapamycin and PBS+IL-33. Data are representative of 2 independent experiments (6 – 8/group). Mann-Whitney U test. *P < 0.05 and **P < 0.01. Rapa, Rapamycin; i.p, intraperitoneal; i.n., intranasal; PBS+PBS, PBS i.p. and PBS i.n; Rapa+PBS, Rapa i.p. and PBS i.n.; PBS+IL-33, PBS i.p. and IL-33 i.n.; Rapa+IL-33, Rapa i.p. and IL-33 i.n.

After demonstrating the induction of mTORC1 activity in bone marrow ILC2s after IL-33 stimulation and the reduction of mTORC1 activity by rapamycin treatment, we investigated this mechanism in ILC2s *in vivo*. The number of ILC2s was similar in all experiment groups (**Figure 4A**), though an increase in pRPS6^+^ ILC2s was observed in mice challenged with IL-33 compared to controls (**Figure 4B**). A reduction of pRPS6^+^ ILC2s was observed in mice pre-treated with rapamycin before IL-33 exposure. The intensity of the pRPS6 signal was increased in IL-33-exposed mice compared to controls (**Figure 4C**) and decreased in mice treated with rapamycin in addition to IL-33 (**Figures 4C, D**).

### 3.5 Decreased ST2 Expression on T_H_ Cells After Rapamycin Treatment

**Figure 5:**
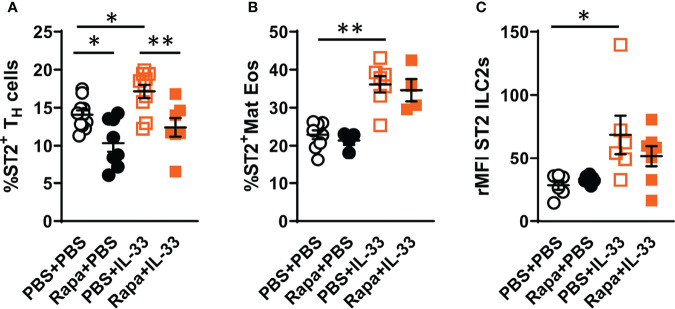
Decreased ST2 expression on bone marrow T_H_ cells after rapamycin treatment and IL-33 challenge. **(A)** Number of ST2^+^ T_H_ cells and **(B)** mature eosinophils. **(C)** ST2 expression on ILC2s shown as relative mean fluorescence intensity (rMFI). Data are representative of 1 – 3 independent experiments (4 – 11/group). Mann-Whitney U test. *P < 0.05 and **P < 0.01. T_H_, T helper cell; ST2=IL-33 receptor; Mat Eos, Mature eosinophil; Rapa, Rapamycin; i.p, intraperitoneal; i.n., intranasal; PBS+PBS, PBS i.p. and PBS i.n; Rapa+PBS, Rapa i.p. and PBS i.n.; PBS+IL-33, PBS i.p. and IL-33 i.n.; Rapa+IL-33, Rapa i.p. and IL-33 i.n.

It has been shown that IL-33 induces mTOR activation through ST2 signaling (30). Therefore, we investigated the effect of rapamycin on ST2 expression on T_H_ cells, mature eosinophils and ILC2s *in vivo*. The number of ST2^+^ T_H_ cells was increased in mice exposed to IL-33 and decreased in mice exposed to rapamycin in addition to IL-33 (**Figure 5A**). Additionally, rapamycin treatment reduced ST2 expression on T_H_ cells in control mice given saline. In contrast to T_H_ cells, rapamycin treatment did not affect the ST2 expression on eosinophils or ILC2s (**Figures 5B, C**). Mice exposed to IL-33 showed an increase in ST2^+^ mature eosinophils and ST2 expression on ILC2s (**Figures 5B, C**).

## 4 Discussion

In this study, the mTORC1 signaling pathway in bone marrow ILC2s was investigated using an IL-33-induced *in vivo* model. Furthermore, the mTORC1 inhibitor, rapamycin, was used to investigate how the treatment affected proinflammatory properties of bone marrow ILC2s *in vivo*. We show for the first time, at the single-cell level, that IL-33 promoted high mTORC1 activity, as measured by the phosphorylation of the known mTORC1 target rpS6 in bone marrow ILC2s both *ex vivo* and *in vivo.* Moreover, this activity was reduced by rapamycin treatment.

There is substantial evidence that IL-5 is a critical cytokine acting on numerous processes in eosinophil biology, driving eosinophilic diseases. Eosinophils develop from CD34^+^ progenitor cells in the bone marrow, where IL-5 promotes terminal eosinophil differentiation and trafficking of bone marrow eosinophils to the airways during different inflammatory conditions (16, 34–36). IL-33 stimulation of bone marrow cultures from naïve WT mice revealed an increased IL-5 production by bone marrow ILC2s and confirmed our previous studies (9, 20, 21). In the current study, we also demonstrated for the first time that the mTORC1 inhibitor rapamycin significantly reduced the number of IL-5^+^ ILC2s in bone marrow cultures stimulated with IL-33. Furthermore, rapamycin also exhibited an effect under basal conditions, as demonstrated by decreased IL-5 production in bone marrow ILC2s in mice given saline.

Extensive evidence indicates that ILCs have tissue-specific functions, and more research is needed to better understand the properties of ILC2s in different tissues during various inflammatory conditions. A lower IL-5 production by lung ILC2s has been reported after rapamycin treatment (30). The same study reported the absence of pRPS6 in sorted lung ILC2s stimulated with IL-33 and rapamycin *in vitro* compared to ILC2s stimulated with IL-33 only (30). We found that rapamycin inhibited IL-5 production by bone marrow ILC2s, thus decreasing the eosinophilic inflammation induced by IL-33. Furthermore, our study demonstrated, *in vivo*, that bone marrow ILC2s act similarly to lung ILC2s during eosinophilic inflammation driven by IL-33.

Assessment of bone marrow eosinophils revealed a decrease in mature eosinophils in IL-33-exposed mice pre-treated with rapamycin compared to mice given IL-33 only. We have previously shown that ILC2s produce large amounts of IL-5 in response to IL-33 which is in contrast to eosinophil progenitors that produce low levels of IL-5 (20). Moreover, we further demonstrate that rapamycin treatment had no effect on IL-5Rα expression on eosinophil progenitors which remained the same in all treatment groups. Together, these data indicate that the lower IL-5 production by bone marrow ILC2s observed in the present study may explain the decreased number of mature eosinophils in mice treated with rapamycin. Our data suggest that the mTORC1 signaling pathway is critical for eosinophilic inflammation and type 2 responses mediated by IL-5-producing ILC2s in the bone marrow. A decrease in airway and mature bone marrow eosinophils in mice treated with rapamycin and IL-33, compared to IL-33 only, indicates that rapamycin may affect the maturation of eosinophils in the bone marrow.

The chemokine receptor CCR3 is highly expressed on eosinophils and contributes to the accumulation and activation of eosinophils (15). Expression of CCR3 on mature eosinophils in the bone marrow remained unchanged in mice exposed to IL-33 compared to control mice, but was reduced in IL-33-exposed mice pre-treated with rapamycin compared to mice given IL-33 only. A reduction in mRNA levels of CCR3 in *in vitro* differentiated bone marrow eosinophils after rapamycin treatment have previously been reported, thus suggesting a cell-intrinsic effect of rapamycin (29). In addition to its role in eosinophil maturation, IL-5 primes eosinophils for migration to sites of inflammation by upregulating CCR3 on eosinophils. Besides a potential cell-intrinsic effect on eosinophils from rapamycin treatment, the decrease in IL-5^+^ ILC2s in bone marrow cultures may indicate that rapamycin affected eosinophil recruitment to the airways by downregulating CCR3. Moreover, a decrease in the CCR3 ligand CCL24/eotaxin-2 was observed in the airways in mice exposed to rapamycin in addition to IL-33 compared to IL-33 only, further suggesting that the recruitment might be affected. Taken together, reduced expression of CCR3 on eosinophils and CCL24/eotaxin-2 in the airways could potentially explain the decrease in airway eosinophilia observed in mice given rapamycin in addition to IL-33.

Our findings confirm previously reported reduced airway eosinophilia after rapamycin treatment in IL-33-exposed mice (30). Furthermore, several studies have also reported a decrease in eosinophils in the airways of mice treated with rapamycin in OVA-induced allergic eosinophilic inflammation (29, 31, 37). One study reported paradoxical effects of rapamycin in HDM-induced airway eosinophilia (28). In that study, rapamycin decreased eosinophilia when administered together with HDM, but increased eosinophilia when administered after an established HDM-induced inflammation. These findings highlight the complexity of mTOR activation and the need for further investigation into how rapamycin affects eosinophilic inflammation.

Several immune cells express ST2, including ILC2s, T_H_ cells and eosinophils (38, 39). We have previously shown that bone marrow ILC2s, T_H_ cells and mature eosinophils all respond to IL-33 by upregulating the ST2 receptor in mice exposed to IL-33, papain or HDM compared to control mice (9, 20, 21). Moreover, it has been proposed that IL-33 induces mTOR activation through ST2 signaling in experiments performed on a murine T_H_2 clone (30). However, little is known about how mTOR inhibition affects ST2 expression on immune cells in the bone marrow. In the present study, we demonstrated a decrease in ST2^+^ T_H_ cells in mice pre-treated with rapamycin in both IL-33-exposed mice and control mice, whereas ST2 expression was not affected on mature eosinophils or ILC2s in any of the examined groups. Further kinetic studies are needed to investigate the clinical impact of decreased ST2 expression after rapamycin treatment. Kinetic studies would also reveal whether ST2 expression on eosinophils and ILC2s varies at other time points or if the altered ST2 expression after rapamycin treatment is unique for T_H_ cells. In contrast to ILC2s which all express ST2, approximately 15% of all bone marrow T_H_ cells of control mice expressed ST2. The difference in ST2 expression may explain the absence of rpS6 phosphorylation after IL-33 stimulation in T_H_ cells. A detailed characterisation of T cell subsets was not addressed in the current study. Thus, in future studies, it would be interesting to determine the effects of rapamycin treatment on ST2 expression in both T_H_2 cells and T regulatory cells and assess the downstream effects during IL-33-induced inflammation.

Clinical trials of anti-IL-33 therapies for asthma are already underway. For patients who do not respond to currently available pharmacological treatments, it is imperative to identify new disease mechanisms during IL-33 inflammation (40, 41). Indeed, future studies that target both mTOR signaling pathways are warranted to deepen the mechanistic understanding of how the mTOR signaling pathways regulate bone marrow ILC2s during IL-33-driven inflammation.

To conclude, we demonstrated for the first time that bone marrow ILC2s responded to IL-33 *in vivo* with increased mTORC1 activity. Rapamycin treatment decreased IL-33-induced eosinophilic airway inflammation, possibly by inhibiting IL-5-producing bone marrow ILC2s. Collectively, our data support previous studies demonstrating beneficial effects of rapamycin treatment during eosinophilic inflammation. The mTORC1 signaling pathway might be a disease driver and a future therapeutic target in inflammatory diseases including asthma.

## Data Availability Statement

The raw data supporting the conclusions of this article will be made available by the authors, without undue reservation.

## Ethics Statement

The animal study was reviewed and approved by Gothenburg County Regional Ethical Committee (permit numbers 126/14 and 2459/19).

## Author Contributions

EB, JW, CM and MR designed experiments. EB, JW, CM, JC and CC performed experiments. EB analyzed data. EB, JW, CM and MR wrote the manuscript. EB, JW, CM, JC, CC and MR revised the manuscript. EB, JW, CM, JC, CC and MR approved the final manuscript. All authors contributed to the article and approved the submitted version.

## Funding

This research was funded by grants from the Swedish Research Council (202001815), the Swedish Heart and Lung Foundation (20190378) and the VBG Group Herman Krefting Foundation for Asthma and Allergy Research.

## Conflict of Interest

The authors declare that the research was conducted in the absence of any commercial or financial relationships that could be construed as a potential conflict of interest.

## Publisher’s Note

All claims expressed in this article are solely those of the authors and do not necessarily represent those of their affiliated organizations, or those of the publisher, the editors and the reviewers. Any product that may be evaluated in this article, or claim that may be made by its manufacturer, is not guaranteed or endorsed by the publisher.
