# Supplementary material for: Rapamycin Dampens Inflammatory Properties of Bone Marrow ILC2s in IL-33-Induced Eosinophilic Airway Inflammation
Source: Front Immunol. 2022 Jun 3;13:915906. doi: 10.3389/fimmu.2022.915906 (PMC9203889; doi:10.3389/fimmu.2022.915906)
Supplement: Supplementary file 1 [file DataSheet_1.docx]

Supplementary Material


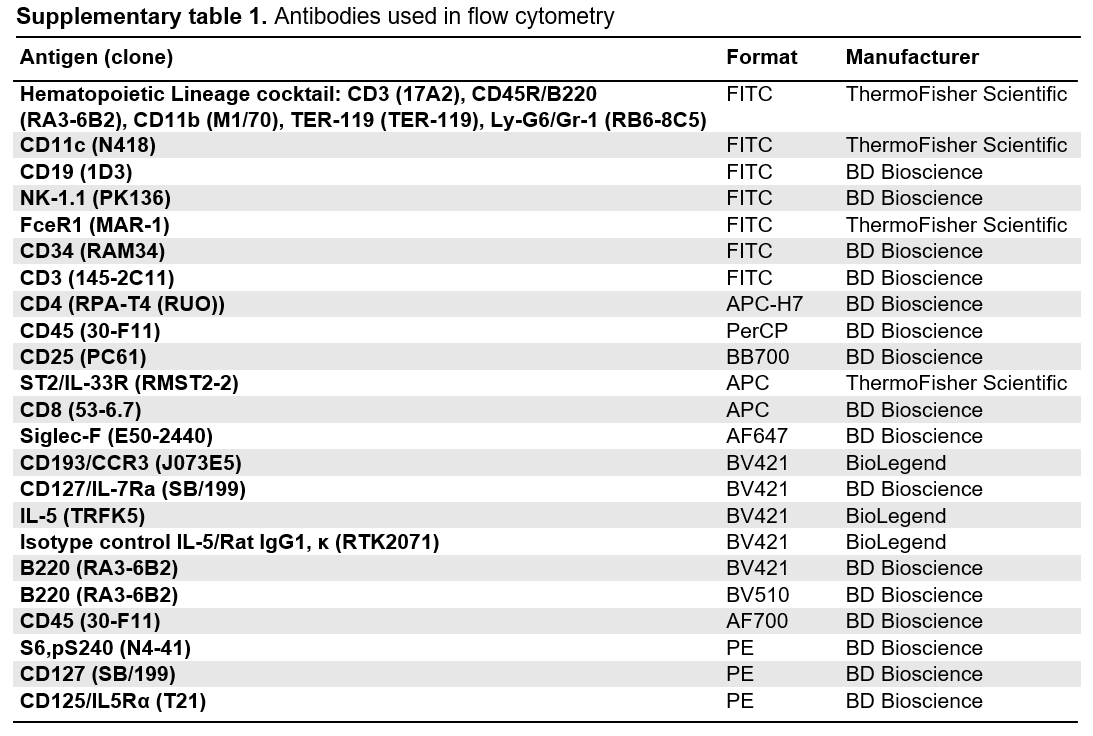


**
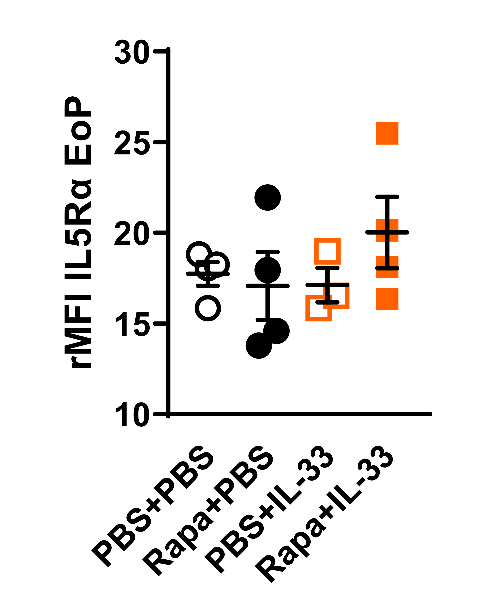
**

**Figure S1.** **Unchanged IL5Rα expression on eosinophil progenitors after rapamycin treatment.** IL5Rα expression on eosinophil progenitors (EoPs) shown as relative mean fluorescence intensity (rMFI). PBS+PBS: PBS i.p. and PBS i.n, Rapa+PBS: Rapa i.p. and PBS i.n., PBS+IL-33: PBS i.p. and IL-33 i.n., Rapa+IL-33: Rapa i.p. and IL-33 i.n.
